# How costly is the first prenatal clinic visit? Analysis of out-of-pocket expenditure in rural Sri Lanka - a country with free maternal health care

**DOI:** 10.1186/s12913-021-07005-y

**Published:** 2021-09-16

**Authors:** Sajaan Praveena Gunarathne, Nuwan Darshana Wickramasinghe, Thilini Chanchala Agampodi, Indika Ruwan Prasanna, Suneth Buddhika Agampodi

**Affiliations:** 1 Department of Community Medicine, Faculty of Medicine and Allied Sciences, Rajarata University of Sri Lanka, Saliyapura, 50008 Sri Lanka; 2 Department of Economics, Faculty of Social Sciences and Humanities, Rajarata University of Sri Lanka, Mihintale, 50300 Sri Lanka

**Keywords:** First prenatal clinic visit, Free health, Out-of-pocket expenditure, Pregnancy, Rajarata pregnancy cohort

## Abstract

**Background:**

This study aimed to determine the magnitude of and factors associated with out-of-pocket expenditure (OOPE) during the first prenatal clinic visit among pregnant women in Anuradhapura district, Sri Lanka, which provides free maternal healthcare.

**Methods:**

The study design was a cross-sectional study, and the study setting was 22 Medical Officers of Health (MOOH) areas in Anuradhapura District, Sri Lanka. Data of 1389 pregnant women were analyzed using descriptive statistics and non-parametric tests.

**Results:**

The mean OOPE of the first prenatal clinic visit was USD 8.12, which accounted for 2.9 and 4.5% of the household income and expenditure, respectively. Pregnant women who used only government-free health services (which are free of charge at the point of service delivery) had an OOPE of USD 3.49. A significant correlation was recorded between household expenditure (r_s_ = 0.095, *p* = 0.002) and the number of pregnancies (r_s_ = − 0.155, *p* < 0.001) with OOPE. Education level less than primary education is positively contributed to OOPE (*p* < 0.05), and utilizing government-free maternal health services lead to a decrease in the OOPE for the first prenatal clinic visit (p < 0.05).

**Conclusion:**

Despite having free maternal services, the OOPE of the first prenatal clinic visit is high in rural Sri Lanka. One-fifth of pregnant women utilize private health services, and pregnant women who used only government-free maternal health services also spend a direct medical cost for medicines/micronutrient supplements.

## Background

The concept of out-of-pocket expenditure (OOPE) on health care refers to the payments made by individuals and families for medical services [1] which is widely defined as ‘direct payment for the cost of care’ [2–4]. The World Bank defines OOPE as ‘any direct payout by households, including gratuities and in-kind payments, to health care providers of pharmaceuticals, therapeutic appliances, and other goods and services whose primary intent is to contribute to the restoration or enhancement of the health status of individuals or population groups’ [5–8]. OOPE is considered a vital issue in the global health agenda, as it impedes enhancing the population health most effectively under the society’s available resources and competing needs [9].

OOPE exerts its negative consequences at the individual, family, community, and national levels [10]. OOPE on health reported as a leading factor in maternal mortality [8, 11, 12], and countries with high OOPE report a higher maternal mortality ratio [8, 11]. Poverty, indebtedness, and unbearable household budget due to the catastrophic health expenditures events [9, 13, 14], changing the household consumption behavior [15], misuse of health care resources, reducing utilization of health care [10, 16–22], barriers to accessing health care facilities [13, 16, 23, 24] are some of the other implications [10]. Further, the failure of the aims in the health care system is caused by high OOPE, especially in the countries which have imposed free health facilities at the time of service delivery [13, 15, 16, 23, 25]. Also, high OOPE is a barrierto achieve global health-related goals Universal Health Coverage [4] and the Sustainable Development Goals (SDGs)-goal 3, and Good Health and Wellbeing [26, 27].

The influence of OOPE is further highlighted in the sub-sectors of essential health care, such as in the provision of maternal health services [28, 29]. Generally, OOPE in maternal health is referred to all the direct monetary expenditures incurred by a woman or her family during the pregnancy [25, 30]. The OOPE in maternal health can divide into *direct medical expenses* and *direct non-medical expenses*.

Direct medical OOPE includes the consultation cost, laboratory tests, treatments, drugs, and consumables. It also includes charges for admission, hospital stay, investigations, and treatment and management [10]. Direct non-medical OOPE is comprised of the cost of traveling, food and lodging of the pregnant mother or family members/accompanying person, accommodation, and cost for servant/maid for household work [10, 14, 15]. In addition, informal payment is considered one of the major direct non-medical payments in developing countries [31–34]. The informal payments are made to receive quality care, avoid waiting in the queue, or receive other health care [32]. High OOPE is a barrier to affordability and accessibility creating inequities in maternal health [28, 29]. Studies performed in low and middle-income countries (LMICs) such as India [35], Pakistan [36], Zambia [37], and Ghana [38] revealed that OOPE is a significant barrier to optimal maternal care.

Sri Lanka exerts free government health services since the early 1900s [39]. The country accommodates free health services by allocating an annual average budget of around 163,079.6 million rupees during the last 3 years, which accounted for 1.6% of the gross domestic product in 2019 [40]. However, it is reported as OOPE accounts for more than 35% of current health expenditure, which has remained from 2007 to 2017 [41, 42]. At present, Sri Lankan maternal health services are well developed and accessed by pregnant women via the public (to a more considerable extent) and the private health system. However, over-medicalization is a significant problem in the Sri Lankan maternal health care service provision, which is in the fourth phase of the obstetric transition [39, 43].

Sri Lanka is a leading country among the LMICs when considering the SDG targets related to maternal and child health. This country has a unique public health system delivering maternal care through a well-established network of primary health care officers. This is unique because it provides domiciliary care and clinic-based service free of charge through grass-root level health officers Public Health Midwives (PHMM). Sri Lankan health system cover 95% of pregnant women through a well-established pregnancy registration system [44]. However, there is a lack of evidence in terms of OOPE in maternal health in Sri Lanka, and all the available evidence on OOPE was related to other aspects of health care. Further, the evidence-based OOPE in its special events (clinic visits, health care seeking for maternal morbidities and other special health-seeking, etc.) is lacking. A detailed assessment of OOPE on maternal care in a country with high health system standards would be valuable in generating global level evidence on the manifestations of OOPE. .

This study aimed to explore the magnitude and associated factors of OOPE among pregnant women during the first prenatal clinic visit in rural Sri Lanka. Therefore, we explored the following two questions that remain unanswered in the rural Sri Lankan context: What are the magnitudes of OOPE of the first prenatal clinic visit of rural Sri Lanka? What are the associated factors of OOPE in the first prenatal clinic visit?

## Methods

This study was a part of a large cohort survey of pregnant women living in the Anuradhapura district, Sri Lanka, Rajarata Pregnancy Cohort (RaPCo). The RaPCo study is a prospective cohort study, mainly conducted to identify the implications of socio-economic, demographic characteristics, and maternal mental health status on pregnancy and newborn outcomes [44]. An economic evaluation under the RaPCo study has been conducted to examine the economic burden of OOPE, productivity loss/cost of pregnant women, and the impact of the COVID-19 pandemic on the household economy of pregnant women in rural Sri Lanka [7]. The complete economic evaluation was a follow-up study that was carried out throughout the pregnancy period. The present study is a part of the sub-component (economic burden of OOPE) of the economic evaluation of the RaPCo study [7, 44].

### Study design and setting

The present study is a cross-sectional study in a cohort of pregnant women enrolled in the RaPCo study during the first trimester and held in Anuradhapura, the largest district in Sri Lanka (7179 km^2^). The recorded total population is 902,930, and 92.7% of them live in rural areas. Agriculture is their primary source of revenue with a median household income of LKR 41,629.00 (United States Dollar [USD] 285.91) per month [45], and Sinhalese are the leading ethnic group (90.7%) [46]. In the Anuradhapura district, health expenditure consists of 42% private, 22% central government, and 36% other government sources [47, 48].

The services for pregnant women are offered via the Medical Officer of Health (MOH), and the Anuradhapura district has 22 MOH areas. The district is divided into 275 public health midwife (PHM) areas, with each PHM area having a population of 1500–4000. The number of pregnant women registered in the area in 2015 was approximately 17,000, and the number of live births was 15,376 [44]. The study protocol of the RaPCo has published further details on the study setting, including MOH areas [44].

### Study participants

The study population of the RaPCo study included all pregnant women residing in the Anuradhapura district from July to September 2019 with the following eligibility criteria: first; pregnant women registered in the “pregnant mothers’ register” of PHMM and visiting antenatal field clinics in the Anuradhapura district, second; permanent residence in the Anuradhapura district for the year ahead, third; period of amenorrhea (POA)/gestational age (GA) less than 12 weeks by the time of recruitment. Pregnant women planning to leave the study area for or after childbirth and pregnant women with uncertain dates were excluded from the study. Pregnant women were recruited with the assistance of the PHMM of each MOH area at the special clinics organized by the cooperation of the research team and the divisional level public health officers. All MOOH and PHMM were informed of the study objectives and the data collection process.

Considering the sensitive nature of data that included the in-depth financial details collected for this investigation, only the women who volunteered to provide such information were chosen to participate in this study. Written consent was sorted from the participants, and ethical and administrative clearance was obtained before recruitment.

### Definitions and measures

The OOPE associated with the first prenatal clinic visit, known as the ‘booking visit,’ was assessed in detail during this study. In Sri Lankan maternal health services, the ‘booking visit’ is the first clinic visit by a pregnant mother. These services are freely available to any expectant mother throughout the country’s public health sector [49]. During this visit, all pregnant women are examined by a medical officer, and generally, all relevant investigations are done in the government healthcare sector, free-of-charge, under several objectives^1^ [49]. Private health services are also available, where pregnant women can channel a specialist in the private sector. This visit includes the cost of consultation, laboratory investigations, and medicines/supplements.

To assess the OOPE associated with the booking visit, the OOPE was computed under two main categories: direct medical cost and direct non-medical cost.

- Direct medical cost (cost for consultation and cost for medicines/micronutrient supplements)
- Direct non-medical cost (cost for traveling, costs for foods and refreshments, the cost for accompanying person, and other costs)

All recruited women were provided with facilities for basic investigations free-of-charge in the RaPCo study; hence, this analysis did not include any laboratory investigation cost.

### Data collection

The study instruments included an interviewer-administered questionnaire on socio-demographic and household economic data and a self-administered questionnaire on pregnancy-related economic data. The study tools were pre-tested and edited according to the pre-tested sample’s answers, comments, and suggestions. Both surveys were conducted during the first trimester.

The participants were provided with the self-administered questionnaire to be filled at home. In addition to explaining how to complete the self-administered questionnaire, an information leaflet was attached, stating the study’s overall objective and instructions to complete— to improve the data credibility. Pregnant women were asked to keep pregnancy-related expenditures in a diary before filling the questionnaire, and telephone reminders were made to complete the questionnaires within 2 weeks.

### Data analysis

Data were manually entered in Microsoft Office Excel and imported into SPSS for analysis. Data entry was followed by data cleaning to identify incompatible entries and missing data. All the incompatible entries were checked manually before eliminating them from the database. The Statistical Package for Social Sciences (IBM SPSS Statistics 21) was used for data analysis.

All the income and expenditure-related data were collected in Sri Lankan Rupees (LKR). In the data management stage, the monitory values were converted from LKR to United States Dollar (USD), according to the rates of July 2019, i.e., USD 1 = LKR 176.38 [50].

Sample characteristics were reported using descriptive statistical measures; mean (SD), median (IQR), mode, and frequencies. Descriptive statistics helped to summarize the magnitude of OOPE. Further, we presented the proportion of OOPE over household income and expenditure. The total OOPE was presented in terms of different income quintiles. The OOPE occurred for pregnant women who used private medical care and government-free health facilities (which are free of charge at the point of service delivery) using descriptive statistical measures. To analyze the associated factors, we performed the Kolmogorov-Smirnov test to assess the distribution of the variable OOPE. The test result disclosed the data are not normally distributed (*p* < 0.05). Therefore, we used non-parametric tests to assess statistical significance and conducted the Mann-Whitney U test and the Kruskal-Wallis H test for the categorical variables. The continuous variables were analyzed via the Spearman Rank Correlation. Further, we performed a Multiple Linear Regression Model (MLRM) to find the impact of associated factors on OOPE. The study sample used in the MLRM is adequate to get an excellent prediction according to the sample Size recommendations at Selected Levels of squared population multiple correlation coefficients (R Square) for performing MLRM [51].

## Results

### Sample characteristics

All the pregnant women (*n* = 3367) of the RaPCo study were invited for the present study and 1389 pregnant women were participated with a response rate of 41.3%. All the sample characteristics were presented in Table 1. The mean (Standard Deviation [SD]) age of the participants was 28.3 (5.5) years. The sample’s leading ethnic group was Sinhalese (*n* = 1184, 89.8%), and the main religion was Buddhism (*n* = 1172, 88.8%). Most pregnant women have studied up to Grade 11 (*n* = 641, 48.8%). In the sample, 78.8% of pregnant women were housewives; not engage in any income-generating activities (*n* = 1094), and among the employed group, 38.4% (*n* = 112) were working in the government sector.

**Table 1:** Characteristics of the surveyed sample

Characteristics/measurement		n	Percentage (%)/statistics
Ethnicity	Sinhalese	1184	89.8%
	Tamil	6	0.4%
	Moor	124	9.4%
	Malay	1	0.1%
	Other	4	0.3%
Religion	Buddhist	1172	88.8%
	Catholic/Christian	18	1.4%
	Hindu	4	0.3%
	Islam	126	9.5%
Education level (in school)	Less than primary education (< grade 5)	10	0.8%
	Up to primary education	2	0.2%
	Between grades 5 to 11	116	8.8%
	Up to ordinary level examination (O/L)	641	48.8%
	Up to advanced level examination (A/L)	544	41.4%
Employment status	Employed	295	21.2%
	Housewives (do not engage in any income-generating activities)	1094	78.8%
Employment sector	Government	112	38.4%
	Semi-government	15	5.1%
	Private	79	27.1%
	Other	86	29.4%
Age (in years)	Mean (SD)	1389	28.3 (5.5)
	Median (IQR)		28 (25–32)
No. of pregnancies	Mean (SD)	1389	2.1 (1.1)
	Median (IQR)		2 (1–3)
	1	510	36.7%
	2	416	29.9%
	3	335	24.1%
	4 and more	128	9.3%
Period of Amenorrhea (POA)-at the time of recruitment (in weeks)	Mean (SD)	1389	9.2 (3.1)
	Median (IQR)		9 (7–10)
	< 6 weeks	174	13.0%
	6–8 weeks	484	36.2%
	8–10 weeks	372	27.8%
	10–12 weeks	173	12.9%
	> 12 weeks	135	10.1%
Mother’s monthly income (USD)	Mean (SD)	295	154.82 (126.20)
	Median (IQR)		141.74 (85.04–203.54)
Monthly household income (USD)	Mean (SD)	1389	283.24 (220.99)
	Median (IQR)		226.78 (170.09–323.17)
Monthly household expenditure (USD)	Mean (SD)	1362	184.81 (119.20)
	Median (IQR)		163.00 (111.12–230.47)

Most pregnant women were in their first pregnancy (*n* = 510, 36.7%). The mean (SD) duration until pregnancy confirmation was 6.4 (2.7) weeks. Public transport was the primary transportation mode for accessing health care facilities (*n* = 895, 68.9%), and most pregnant women (*n* = 784, 61.3%) preferred to use government health facilities.

The mean (SD) and the median (Inter Quartile Range [IQR]) monthly income of the employed pregnant women were USD 154.82 (126.20) and USD 141.74 (85.04–203.54), respectively. The mean (SD) of the monthly household income and the expenditure were USD 283.24 (220.99) and USD 184.81 (119.20), respectively. The corresponding median (IQR) values were USD 226.78 (170.09–323.17) and USD 163.00 (111.12–230.47).

### The magnitude of OOPE for the first prenatal clinic visit

#### Breakdown of OOPE- direct medical and non-medical cost

The mean (SD) OOPE for the first prenatal clinic visit was USD 8.12 (16.47). Compared to monthly household income and expenditure, the total OOPE for the first prenatal clinic visit was approximately 2.9% of revenue and 4.5% of the spending. This was calculated by assuming that the monthly household income and the spending do not change in the short run since a shorter period was considered for the present study.

The breakdown of OOPE in the first prenatal clinic visit was reported in terms of the direct medical and direct non-medical cost and according to the health facility used in Table 3.

The mean (SD) direct medical OOPE of the first prenatal clinic visit was USD 16.26 (20.45), and it was 68.2% of the total OOPE; thus, it is almost twice the value of the direct non-medical cost. Among the direct medical expense, the cost share for consultation (Mean = USD 16.41, SD = 17.10) was the highest, followed by the cost for medicines/micronutrient supplements (Mean = USD 7.72, SD = 10.22).

**Fig. 1:**
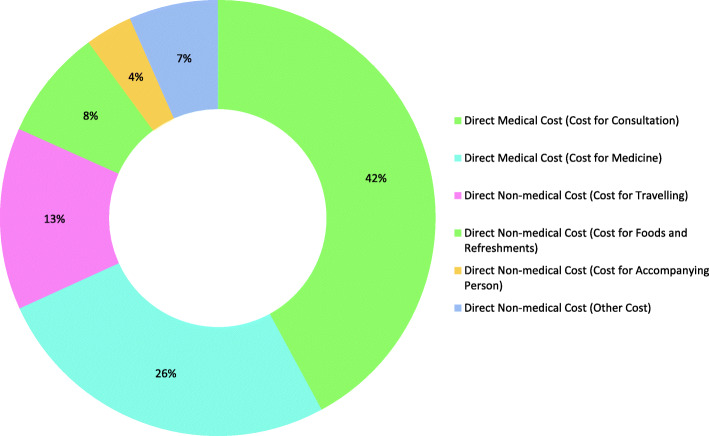
Breakdown of OOPE

Figure 1 presents the breakdown of all expenditures and the composition of direct medical and direct non-medical costs during the first trimester of the pregnant women in the sample. It highlights that the costs for consultation had the highest proportion of OOPE among all cost categories and accounted for 42.1%. In contrast, the cost for the accompanying person is the minor share, 3.5% of the total OOPE.

Among the surveyed sample, 343 pregnant women (24.7%) financed health care by spending money kept for their everyday transaction purposes, and 191 pregnant women (13.8%) have withdrawn money from their savings. Only one mother from the entire study sample owned a health insurance policy, while others were financed by informal loans with high-interest rates (*n* = 14, 1.0%) and selling assets such as jewelry (*n* = 7, 0.5%).

#### OOPE among different income quintiles

Table 2 presents the OOPE among different income quintiles.

**Table 2:** OOPE among different income quintiles

Income Quintile (USD)		OOPE (USD)		n (%)	OOPE on household Income (%)	OOPE on household cost (%)
		Mean (SD)	Median (IQR)			
1	< 170.08	8.79 (13.83)	2.83 (1.13–11.06)	383 (27.6%)	4.4%	3.6%
2	170.09–215.44	10.30 (13.76)	3.12 (1.13–17.29)	192 (13.8%)	4.5%	5.1%
3	215.45–255.13	15.28 (24.13)	3.40 (1.13–21.97)	277 (19.9%)	4.8%	5.9%
4	255.14–368.52	10.31 (18.86)	2.83 (0.85–11.34)	282 (20.3%)	2.5%	3.9%
5	> 368.53	10.12 (18.32)	3.01 (1.13–16.44)	255 (18.4)	1.3%	4.1%

The lowest income groups’ (< USD 170.08 and USD 170.09–215.44) OOPE for the first prenatal clinic visit were USD 8.79 and USD 10.30, 4.4 and 4.5% monthly household income, respectively.

Among the pregnant women in the lowest income quintile, 61.3% of expenditure was direct medical expenditure; the mean (SD) OOPE were USD 14.56 (7.41) for consultation and USD 5.64 (9.20) for medicines, which accounted for 37.9 and 23.4% of total OOPE for the first prenatal clinic visit. The direct non-medical expenditure accounted for 38.7% within the lowest income quintile.

### OOPE in government-free health services and private health services

Table 3 indicates the direct medical and direct non-medical expenditure incurred for the first prenatal clinic visit among pregnant women who used only government-free health services and private health services. The majority (*n* = 1100, 79.2%) in the study sample used only the government’s free maternal health services. There is a statistically significant difference in direct non-medical OOPE between pregnant women who used only the government’s free health services and those who used private health services (U = 52,890.0, |*z*| =7.9). Even among the pregnant women who used free maternal health services, the OOPE was USD 3.49 (SD = 6.53). It included all the direct non-medical expenditures and costs for medicines/micronutrient supplements as the direct medical cost. The mean (SD) direct medical OOPE in terms of cost for medicines/micronutrient supplements for the first prenatal clinic visit was USD 2.82 (6.17).

**Table 3:** OOPE of the first prenatal clinic visit (for government-free and private health facilities)

Cost Category		The cost incurred using only government free health care (USD)			The cost incurred in using private health care (USD)			OOPE of the total sample		
		n (% of the sample)	Mean (SD)	Median (IQR)	n (% of the sample)	Mean (SD)	Median (IQR)	n (% of the sample)	Mean (SD)	Median (IQR)
Direct Medical OOPE	Cost for consultation	–	–	–	289 (20.8%)	16.41 (17.10)	14.74 (11.34–17.01)	289 (20.8%)	16.41 (17.10)	14.74 (11.34–17.01)
	Cost for medicine/micronutrient supplements	189 (13.6%)	2.82 (6.17)	0.85 (0.57–2.55)	198 (14.3%)	12.27 (11.16)	9.07 (3.97–17.01)	387 (27.8%)	7.72 (10.22)	2.83 (0.62–11.34)
Direct non-medical OOPE	Cost for travelling	521 (37.5%)	1.58 (2.80)	0.85 (0.57–1.70)	172 (12.4%)	4.05 (5.84)	1.90 (1.13–4.40)	693 (49.9%)	2.19 (3.93)	1.13 (0.57–1.7)
	Food and refreshment	440 (31.7%)	1.27 (1.30)	1.13 (0.57–1.56)	154 (11.1%)	2.45 (2.86)	1.70 (1.13–2.83)	594 (42.8%)	1.58 (1.90)	1.13 (0.57–1.70)
	Cost for accompanying person	204 (14.7%)	1.22 (1.47)	0.85 (0.57–1.13)	58 (4.2%)	2.44 (2.41)	1.70 (1.13–2.83)	262 (18.9%)	1.49 (1.79)	0.85 (0.57–1.70)
	Other costs	156 (11.2%)	2.94 (4.71)	0.71 (0.57–3.72)	32 (2.3%)	8.73 (12.16)	2.83 (0.88–12.76)	188 (13.5%)	3.93 (6.90)	0.85 (0.57–5.66)
**Total (OOPE)**		**1100 (79.2%)**	**3.49** **(6.53)**	**1.70** **(0.85–3.54)**	**289 (20.8%)**	**29.99** **(24.09)**	**24.38** **(17.31–34.58)**	**1389 (100%)**	**8.12 (16.47)**	**2.95 (1.13–16.02)**

**Fig. 2:**
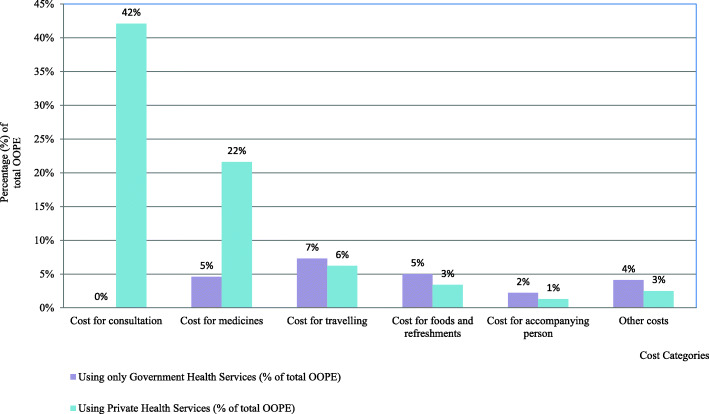
Percentage of cost categories out of the total OOPE by the healthcare facility used

Figure 2 illustrates the share of different cost categories on OOPE according to the health care facilities used. All the direct medical costs were high among the pregnant women who used government and private health services, and the share of all direct non-medical costs was high among pregnant women who used government health facilities only.

### Factors associated with OOPE

Table 4 demonstrates the associated factors of OOPE for the first prenatal clinic visit. There is a statistically significant positive correlationbetween OOPE and monthly household expenditure, and a statistically significant negative correlation between OOPE and the number of pregnancies.

**Table 4:** Associated factors of OOPE

Performed Test	Variable	Groups Tested	Mean (SD) OOPE [USD]	Median (IQR) OOPE [USD]	Test Statistics
Mann-Whitney U Statistics	Employment status	Employed (n_1_ = 295)	13.64 (12.63)	14.17 (1.13–19.84)	U = 92,939.5 (|*z*| =0.263)
		Housewives (not engage in any income-generating activities) (n_2_ = 1094)	17.02 (22.16)	14.46 (1.42–23.96)	
Kruskal-Wallis H Test	Employment sector	Government (n_1_ = 112)	12.61 (11.61)	13.61 (0.85–18.71)	*x*^2^=4.266 (*p* = 0.234)
		Semi-government (n_2_ = 15)	10.09 (7.37)	8.5 (5.67–16.44)	
		Private (n_3_ = 79)	17.20 (13.94)	17.01 (3.4–24.66)	
		Other (n_4_ = 86)	12.56 (13.08)	11.34 (0.91–17.38)	
	Ethnicity	Sinhalese (n_1_ = 1184)	16.32 (21.18)	14.17 (1.13–22.68)	*x*^2^=1.926 (*p* = 0.588)
		Tamil (n_2_ = 6)	12.57 (9.21)	17.01 (9.50–20.98)	
		Moor (n_3_ = 124)	13.73 (13.49)	12.47 (0.85–20.98)	
	Religion	Buddhist (n_1_ = 1172)	16.37 (21.24)	14.17 (1.13–22.68)	*x*^2^=1.944 (*p* = 0.584)
		Catholic/Christian (n_2_ = 18)	9.30 (7.50)	12.76 (1.98–14.17)	
		Hindu (n_3_ = 4)	17.86 (1.20)	17.86 (17.01–18.71)	
		Islam (n_4_ = 126)	13.73 (13.49)	12.47 (0.85–20.98)	
	Education level	Less than primary education (n_1_ = 10)	30.62 (48.49)	9.78 (1.00–60.24)	*x*^2^=3.157 (*p* = 0.532)
		Between grade 5 to 11(n_3_ = 116)	17.45 (13.40)	17.01 (4.12–22.96)	
		Up to ordinary level (n_4_ = 641)	16.28 (21.84)	14.17 (1.13–23.39)	
		Up to advanced level (n_5_ = 544)	15.23 (19.49)	14.17 (1.13–20.41)	
	Period of Amenorrhea (at the time of recruitment)	< 6 weeks (n_1_ = 174)	14.85 (14.01)	14.17 (1.13–19.84)	*x*^2^=1.000 (*p* = 0.910)
		6–8 weeks (n_2_ = 484)	17.10 (24.15)	14.6 (1.13–21.54)	
		8–10 weeks (n_3_ = 372)	16.89 (22.42)	14.74 (1.56–25.23)	
		10–12 weeks (n_4_ = 173)	13.41 (13.28)	12.62 (1.13–19.84)	
		> 12 weeks (n_5_ = 135)	15.05 (16.25)	14.17 (1.13–20.41)	
Spearman Rank Correlation	Number of pregnancies^*^				*r*_*s*_ = −0.155 (*p* = 0.000)
	Age of the mother				*r*_*s*_ = 0.028 (*p* = 0.378)
	Mother’s monthly income level				*r*_*s*_ = 0.012 (*p* = 0.861)
	Monthly household income				*r*_*s*_ = −0.002 (*p* = 0.942)
	Monthly household expenditure^*^				*r*_*s*_ = 0.095 (p = 0.002)

^*^Reject null hypothesis: no correlation – since the *p* values are lesser than 0.05

All 17 independent predictors were identified according to the evidence in the literature and purposefully and included in the MLRM at the initial stage. After the several steps, seven independent variables (Pregnant women’s monthly income, monthly household expenditure, MOH area, Ethnicity, Religion, and the economic status due to the poverty line) were eliminated from the model to avoid the multicollinearity issue and to keep the overall significance of the model. The overall model is statistically significant (F = 68.11, *p* < 0.001) and has an R-square value of 0.49. The model does not show the autocorrelation issue as the Durbin-Watson D statistics is 2.01. Table 5 is presented the impact of predictor variables on the dependent variable OOPE.

**Table 5:** MLRM for the impact of associated factors on OOPE

Model		Coefficient (B)	t-statistics	*P*-value	95.0% Confidence Interval for B		Variance Inflation Factor (VIF)
					Lower Bound	Upper Bound	
Constant^*^		27.76	11.698	0.000	23.10	32.41	–
Monthly household income		−8.605E-6	−0.723	0.470	0.00	0.00	2.05
Income quintile (compared to income quintile 1 [< USD 170.08])	Income quintile 2 (USD 170.09–215.44)	0.77	0.693	0.488	−1.41	2.95	1.36
	Income quintile 3^*^ (USD 215.45–255.13)	2.51	2.480	0.013	0.52	4.49	1.51
	Income quintile 4 (USD 255.14–368.52)	1.52	1.427	0.154	−0.57	3.61	1.67
	Income quintile 5 (> USD 368.53)	1.52	1.004	0.315	−1.45	4.48	3.13
Age of the Mother		0.09	1.510	0.131	−0.03	0.21	1.05
Employment status (compared with the Housewives – do not engage in any income generating activities)	Employed	−1.95	−1.424	0.155	−4.63	0.74	2.81
Sector of the employment (compared to the government sector)	Semi-government	−0.45	−0.248	0.804	−3.10	3.10	2.27
	Private	1.91	1.006	0.315	−1.81	5.63	1.77
Educational level (compared with the ‘up to advanced level examination’)	Less than primary^*^ education (<grade 5)	10.89	2.606	0.009	2.69	19.09	1.02
	Up to primary education	−2.78	−0.334	0.738	−19.07	13.52	1.01
	From grade 5 to 11	0.97	0.749	0.454	−1.57	3.50	1.17
	Up to ordinary level examination	−0.01	−0.015	0.988	−1.47	1.45	1.26
Number of Pregnancies		−0.33	−1.128	0.260	−0.91	0.25	1.02
Number of weeks in pregnancy		−0.09	−0.884	0.377	−0.30	0.12	1.01
Used transport method to reach the health facility (compared with using an own/family vehicle)	Public transport	0.226	0.315	0.752	−1.18	1.63	1.01
Type of the health service used (compared with private health facilities)	Government health facilities^*^	−27.090	−33.031	0.000	−28.70	− 25.48	1.02

^*^significant at 0.05 of the*p*-value

The constant term was statistically significant, reflecting that, when all other factors are being constant, the OOPE of the first prenatal clinic visit is USD 27.76. Further, three dummies of predictor variables were statistically significant. Compared with the lowest income quintile (< USD 170.08), the middle-income group (USD 215.45–255.13) spends more of USD 2.51 as OOPE for the first prenatal clinic visit when other factors are constant. Moreover, compared with the pregnant women who had been educated up to the advanced level examination, the pregnant women who had received less than primary education (<grade 5) expended more of USD 10.89 when controlling other characteristics. In addition, pregnant women who utilize government-free maternal health services pay USD 27.09 less when compared with pregnant women who use private health services for the first prenatal clinic visit.

## Discussion

One primary reason for poor maternal and child health outcomes is identified as the higher OOPE associated with health care, especially among the poor, for whom health care access often imposes a considerable financial burden on families [15, 30, 52]. In this context, the present study aimed to comprehensively assess the OOPE of the first prenatal clinic visit—the “booking visit” in pregnancy care in Sri Lanka.

This study used a probability sample representing the whole district. The total OOPE estimated for the first prenatal clinic visit (USD 8.12) could be underestimated because the laboratory investigations for this cohort were provided free of charge by the RaPCo study. The examinations are offered through the free health care system in Sri Lanka. However, some pregnant women prefer attending paid services to minimize travel and the waiting time in public hospitals. Even with a probable underestimation, the OOPE was 4.5% of the total expenditure, and it is almost equal to the household health expenditure share, which was 4.7% in rural Sri Lanka [53]. This is a considerable amount compared to the population for a single clinic visit in the availability of free healthcare services, especially considering its impact on household income and expenditure. Evidence suggests that the OOPE of maternal healthcare can range between 1 and 5% of total annual household expenditure throughout the pregnancy period and increase between 5 and 34% if complications occur. This could lead to a catastrophic expense for poor households in low-income countries in Asia and Africa [54].

The problem’s severity is further emphasized with the reported 4% of OOPE of monthly household income within the lowest income quintiles. This could be challenging since only one mother was using health insurance for financing health care. Still, others had to withdraw from routine transactions/savings from informal loans with high-interest rates and selling assets. According to statistics, society’s poorest section has to pay for health needs from their expenditure, which they keep for basic necessities [15, 16, 23, 30, 55, 56].

Most pregnant women (79.2%) used government-free health services, and 61.3% of them were below the middle-income quintile. Similarly, the literature suggests that the free government healthcare facilities’ usage rates, including inpatient, primary, and preventive care, were highest among the poor [57]. Even though using only government-free maternal health services, pregnant women had an OOPE of USD 3.49. Among them, one-fifth was for the cost of medicine/micronutrient supplements (Folic acid, iron folate, Vitamin C, and calcium supplementation), and the rest was for unavoidable direct non-medical costs. This study did not specifically collect facts regarding the reasons and types of medicine/micronutrient supplements that pregnant women purchased.

Nonetheless, the government prenatal health care services are provided micronutrient supplements and essential medicine for minor ailments free of charge. In that context, OOPE for the cost of medicine/micronutrient supplements indirectly implies either the unavailability of such medicine at the health care facility due to out-of-stock or pregnant women preferring to purchase them from outside due to various reasons. However, this is a vital issue since Sri Lanka exerts free government health services to all citizens [39, 43] and, primarily, the government-financed healthcare in Sri Lanka [58]. Therefore, the avoidable OOPE (direct medical cost) should be zero or at a minimal level in a setting with a free healthcare policy [10, 15, 24, 59–61]. However, available literature of different regions in the world also confirmed that the existence of OOPE with practicing public free health care policy and national-level free health programs in Nepal [1, 62–64], Bangladesh [1, 62, 65], and India [29, 35].

Among the study sample, 20.8% of pregnant women had utilized private health care services and had paid 42.1% of the cost for consultation and 21.6% for the cost for the medicine of the total OOPE. More importantly, 61.6% of pregnant women who used private health services were below the middle-income quintile. The emerging issue here is that (despite having free maternal healthcare services), many people spend high OOPE, unbearable for the low-income families’ household expenses [10, 13, 15, 66–70]. Instead of accessing free government health care, people tend to bear the actual cost of some drugs, investigations, and surgeries, which may place a significant burden on Sri Lanka’s households [39, 71]. This is under- and mal-utilization of the well-developed maternal health care package to catering all requirements for the initial prenatal clinic [49].

The positive correlation between OOPE and household expenditure is oblivious since OOPE acts as an independent health cost category, including medical and non-medical spending, which is in line with the existing evidence [72]. The number of pregnancies reported a negative association with OOPE; a study conducted in India revealed a similar association [36]. The probable reason could be better financial management during pregnancy with previous experience. Further, the middle-income category and educational level less than primary education was positively contributed high OOPE than others. The reason for the expenditure level of the middle-income group is due to the existing 5.9% of the total household expenditure, which was more than the rural Sri Lankan estimates [53]. In addition, the negative contribution on OOPE as using government-free maternal health services is evident since the Sri Lankan health sector is provided services free of charge.

### Limitations

The findings of the present study need to be understood with the following three limitations. First, the present study has a selection bias due to the lower response rate. Second, there may have a recall bias even though we collect data immediately after the first prenatal clinic visit. Third, OOPE estimation is underestimated since the RaPCo study provided laboratory and other investigations free of charge. It may be reduced the cost for laboratory and further investigations for mothers who were expected to use paid private health facilities.

## Conclusion and recommendations

The study provides strong evidence that the reported OOPE of the first prenatal clinic visit is high since the OOPE share of total expenditure in a single event is almost equal to the rural Sri Lankan estimates. The direct medical cost is almost twice more elevated than the direct non-medical cost. A direct medical cost for medicines/micronutrient supplements is incurred by pregnant women who used only the government-free maternal health services. One-fifth of pregnant women utilize private health services despite having free maternal health services.

In this context, it is essential to ponder upon the implementation gaps in free health provision and create a mechanism to increase the utilization for government-free maternal health services to minimize the additional financial burden and further improve the maternal health care provision in Sri Lanka.

## Data Availability

The datasets used and/or analyzed during the current study are available from the corresponding author on reasonable request.

## Abbreviations

OOPE: Out-of-pocket Expenditure
SDGs: Sustainable Development Goals
LMICs: Low and middle-income countries
RaPCo: Rajarata Pregnancy Cohort
USD: United States Dollar
MOH: Medical Officer of Health
PHM: Public Health Midwife
PHMM: Public Health Midviwes
POA: Period of amenorrhea
GA: Gestational Age
LKR: Sri Lankan Rupees
SD: Standard Deviation
IQR: Inter Quartile Range
MLRM: Multiple Linear Regression Model
AHEAD: Accelerating Higher Education Expansion and Development

## Footnotes

1 1. To the provisional establishment of pregnancy (either clinically/with laboratory testing) 2. To assess medical and obstetric risk factors and take necessary actions 3. To assess the psychological/nutritional status and take necessary actions 4. To provide micronutrient supplements (Folic acid, iron folate, Vitamin C, and calcium supplementation) 5. To provide the necessary care and health promotion 6. Screening for anemia, syphilis, and testing for blood group and Rh 7. Check for dental conditions and appropriate referral 8. Heart examination to identify heart diseases [49].
